# Case Report: Capillary Leak Syndrome With Kidney Transplant Failure Following Autologous Mesenchymal Stem Cell Therapy

**DOI:** 10.3389/fmed.2021.708744

**Published:** 2021-07-21

**Authors:** Željka Večerić-Haler, Nika Kojc, Matjaž Sever, Samo Zver, Urban Švajger, Primož Poženel, Katrina Hartman, Tereza Urdih, Gregor Mlinšek, Manca Oblak, Andreja Aleš Rigler, Alojz Ihan, Jadranka Buturović Ponikvar, Philip P. Halloran, Miha Arnol

**Affiliations:** ^1^Department of Nephrology, University Medical Centre Ljubljana, Ljubljana, Slovenia; ^2^Faculty of Medicine, University of Ljubljana, Ljubljana, Slovenia; ^3^Institute of Pathology, Faculty of Medicine, University of Ljubljana, Ljubljana, Slovenia; ^4^Department of Haematology, University Medical Centre Ljubljana, Ljubljana, Slovenia; ^5^Division for Cells and Tissue, Blood Transfusion Centre of Slovenia, Ljubljana, Slovenia; ^6^Institute of Microbiology and Immunology, Faculty of Medicine, University of Ljubljana, Ljubljana, Slovenia; ^7^Division of Nephrology and Transplant Immunology, University of Alberta, Alberta Transplant Applied Genomics Centre, Edmonton, AB, Canada

**Keywords:** mesenchymal stem cell, kidney graft, kidney transplant, kidney graft failure, autologous stem cell, stem cell transplant, kidney graft rejection, capillary leak syndrome

## Abstract

Mesenchymal stem cells (MSCs) have attracted great interest in the field of kidney transplantation due to their immunomodulatory and reparative properties. In registered clinical trials, MSCs have been used before, at the time of, or early after transplantation and have been reported to be well-tolerated with no serious safety concerns. No results are available on the use of MSCs in the late post-transplant period. Here, we present a case report of a severe systemic complication mimicking capillary leak syndrome with ultimate kidney transplant failure after autologous transplantation of MSCs used as rescue treatment of late antibody-mediated kidney allograft rejection.

## Introduction

Mesenchymal stem cells (MSCs) have attracted much attention due to their immunomodulatory properties that can help in alloimmune diseases. Several factors, including immunosuppressive factors, growth factors, extracellular vesicles, and chemokines, contribute to the immunosuppressive mechanisms of MSCs (1). Pilot studies in clinical research with MSCs in kidney transplant recipients (KTRs) aimed to reduce immunosuppressive therapy, induce immune tolerance, treat T-cell rejection, and prevent delayed graft function (2–11). To date, the administration of MSCs in clinical transplantation has been shown to be safe and feasible without serious safety concerns being reported.

Here, we present a case report of a serious adverse reaction in KTR after autologous transplantation of MSCs from bone marrow, which was used as a rescue treatment for resistant antibody-mediated rejection (AMR). The patient was included in the study protocol (ClinicalTrials.gov, number NCT03585855), which was subsequently discontinued due to safety concerns.

## Case Presentation

**Figure 1:**
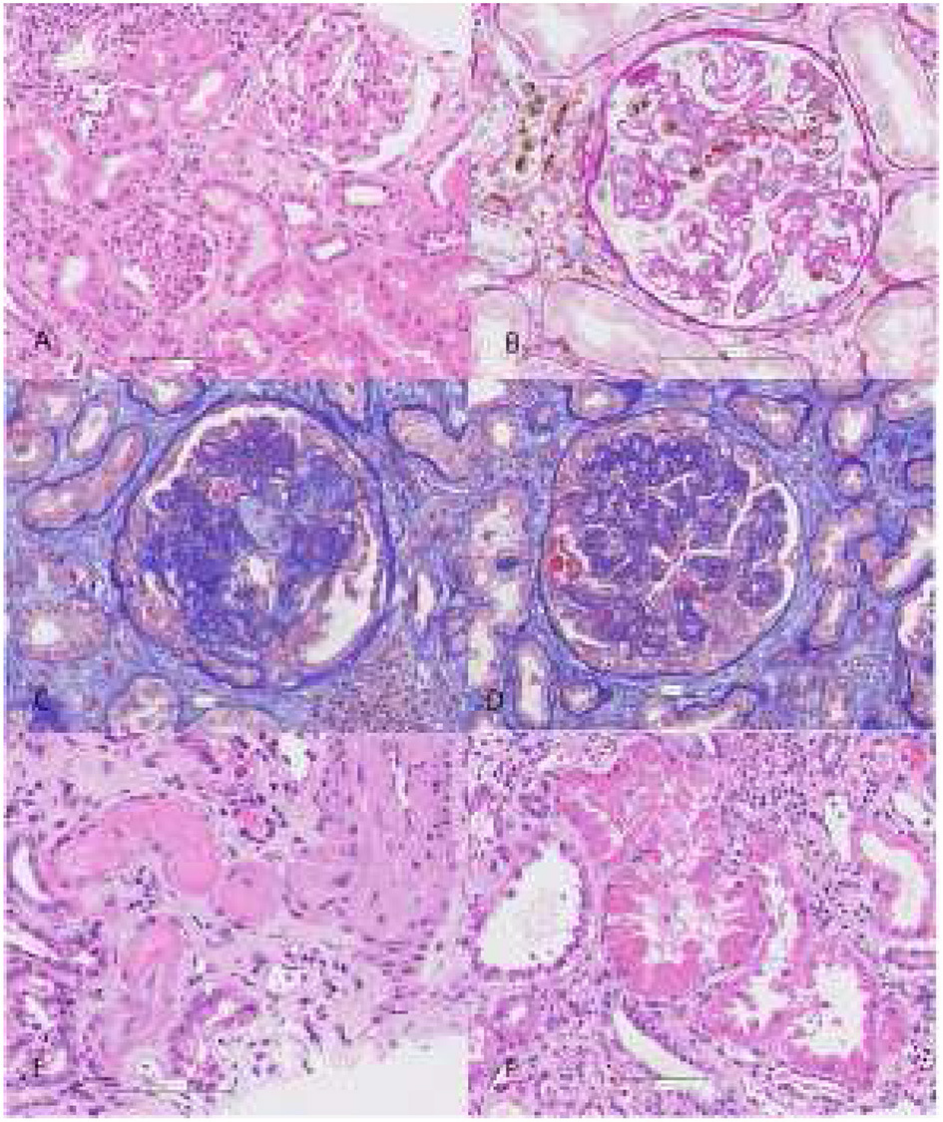
Before application of MSCs (March 2019): chronic active C4d negative antibody-mediated rejection with preserved tubules **(A)**, focal glomerulitis and peritubular capillaritis **(B)**. According to Banff 2017 criteria, transplant kidney biopsy was consistent with chronic active C4d negative antibody mediated rejection: 12% glomerulitis with double contour formation (g1, cg3) in glomeruli, diffuse severe peritubular capillaritis (ptc3), chronic active vascular rejection (v1, cv3) in interlobular arteries without signs of thrombotic microangiopathy, diffuse more than 50% interstitial fibrosis and tubular atrophy with mild mononuclear cell inflammation consisting of lymphocytes, macrophages, and rare plasma cells (i-IFTA 3, ci3, ct3). There was no associated tubulitis. Peritubular capillary basement membrane multilamellation (ptcbm3) was seen by electron microscopy. There were no deposits in tubular basement membrane. Immunofluorescence revealed IgA deposits indicating IgA nephropathy recurrence. 60% of glomeruli were globally sclerotic. 1 of 25 glomeruli showed pseudocrescent formation without PAS positive droplets in podocytes. After application of MSCs (July 2019): glomerular TMA with mesangiolysis **(C)**, diffuse pseudocrescent formation in glomeruli with marked podocytes injury **(D)**, vascular TMA **(E)** and huge tubular injury with resorptive droplets consisted with mottled lysosomes on EM **(F)**. Glomeruli showed advanced double contour formation and segmental sclerosis without apparent glomerulitis, but mesangiolysis was seen in some glomeruli. 20% of glomeruli showed pseudocrescent formation with PAS positive droplets “in podocytes indicating huge podocytes injury”. Peritubular capillaritis was mild and focal. Tubulitis was absent. Tubules show signs of severe tubular injury (attenuation of tubular epithelium with coarse vacuolization, loss of brush border, loss of nuclei) and were filled with large PAS-positive droplets in line mottled lysosomes on electron microscopy. Electron optic dense deposits in tubular basement membrane were detected by EM. Amount of interstitial fibrosis, tubular atrophy, and interstitial inflammation was similar as in previous biopsy. In fibrotic areas, there was mild mononuclear cell inflammation consisting of lymphocytes 75%, macrophages 15%, and plasma cells 10%. No CD105+, CD73+ nor CD90+ cells were found. In small arteries and arterioles, thrombotic microangiopathy with obliteration of vascular lumens, fragmentation of erythrocytes, and fibrinoid necrosis was present. Peritubular capillary basement membrane multilamellation (ptcbm3) was similar as in previous biopsy.

A 26-year-old man with a history of acute lymphoblastic leukemia (ALL) in childhood and end-stage kidney failure due to IgA nephropathy received a deceased donor kidney transplant at the age of 21, mismatched for 3 HLA antigens (one mismatch in HLA-A, HLA-B, and HLA-DR). Two years after kidney transplantation (KTx), an indication biopsy for an increase in serum creatinine (sCr) levels showed a mixed T-cell rejection (Banff 4/IB) and acute AMR with positive donor-specific antibodies (DSA; anti-HLA DQB1 and DQA1) while receiving triple maintenance immunosuppressive regimen (tacrolimus/mycophenolate mofetil/steroid). The rejection was treated with high-dose steroids, antithymocyte globulin, plasmapheresis, intravenous immunoglobulins (IVIg), and rituximab. After 3 years of stable kidney function with sCr in the range between 150 and 180 μmol/L, we observed a progressive deterioration of kidney function with a sCr value of 240 μmol/L and a 24-h proteinuria of 3.4 g/day before entering the study protocol. The kidney biopsy revealed chronic active AMR (Figures 1A,B).

Due to the history of childhood ALL, which was treated with a combination chemotherapy (vincristine, doxorubicine, methotrexate, cyclophosphamide, cytarabine), we first performed a bone marrow aspiration, which showed non-specific reactive changes. The treatment protocols were approved by National Ethic Committee (approval no. 0120-215/2018/4). The written informed consent was obtained from the patient. After completion of the standard of care therapy (including corticosteroids, membrane plasmapheresis, and IVIg), the patient received MSCs therapy according to the study protocol, consisting of 3 x 10^6^ cells/kg, applied at 1-week intervals. Details of the MSCs cultures and criteria for bench release are described in the Supplementary documentation (Supplementary Appendix 1 and Table 1).

**Figure 2:**
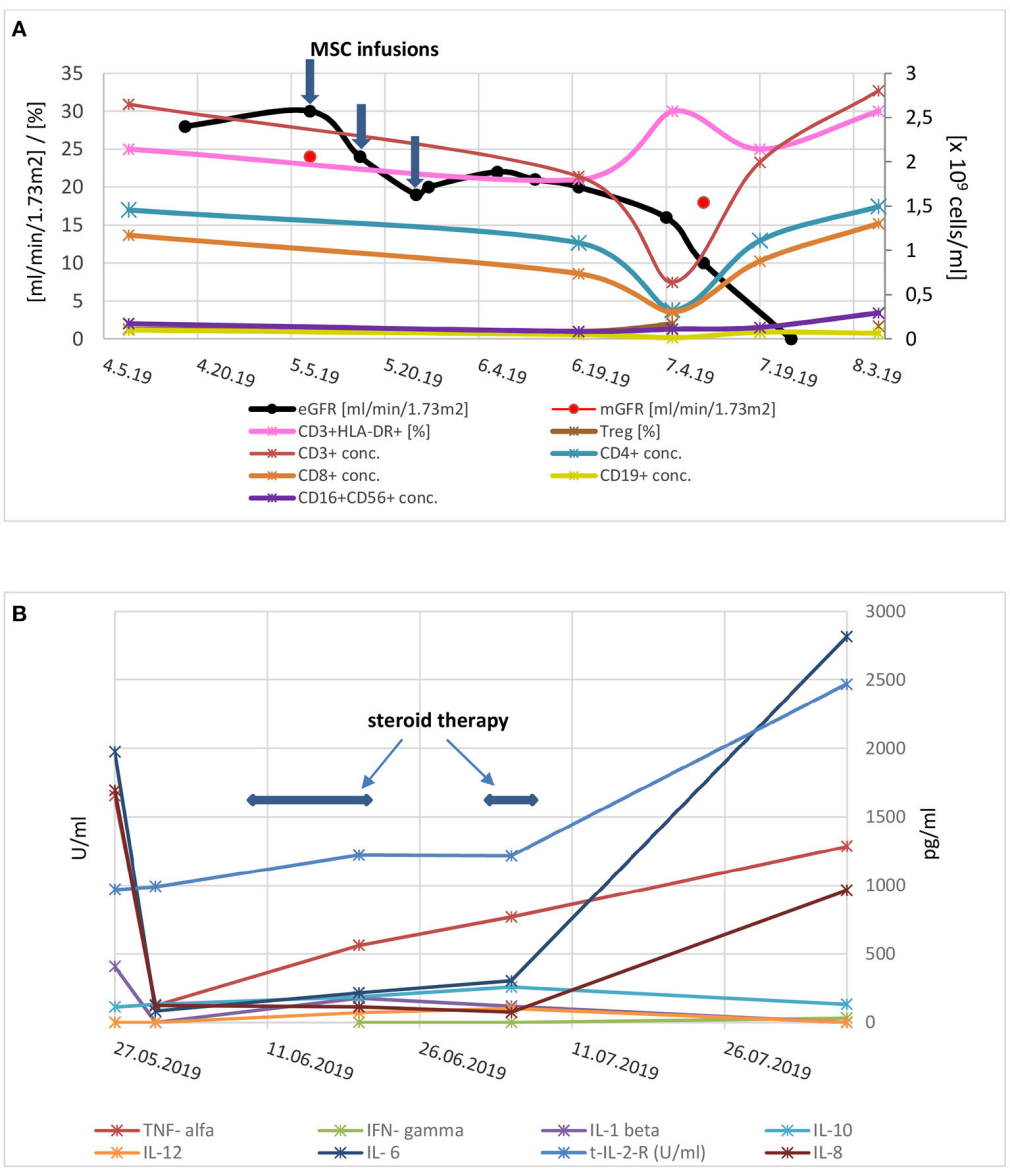
**(A)** Kidney graft function and serum concentrations of lymphocyte populations before and after application of mesenchymal stem cells. Three consecutive applications of mesenchymal stem cells are marked with arrows. eGFR, estimated glomerular filtration rate; mGFR, measured glomerular filtration rate (by Cr-EDTA); conc, concentration; Treg, regulatory T cells. **(B)** Cytokine concentrations after mesenchymal stem cells application (in U/mL for soluble interleukin 2 receptor and in pg/mL for other cytokines). Time frame of corticosteroid treatment is marked with arrows; Legend: TNF, tumor necrosis factor; IL, interleukin; s-IL-2-R, soluble interleukin 2 receptor; IFN, interferon.

After the first dose of MSCs the patient reported short term nausea. One week later after the second dose of MSCs nausea, blepharitis and diarrhea developed, with their remission after 24 h. This was associated with a slight deterioration in kidney function, which we attributed to prerenal causes (Figure 2A). When the patient was referred for the third dose of MSCs (2 weeks after the first dose), the symptoms had completely disappeared. The evening after the third dose of MSCs was administered, the patient was admitted to the emergency department due to abdominal cramps, vomiting, and diarrhea. A further deterioration of the kidney function was observed (sCr of 390 μmol/L), accompanied by newly developed ascites and abdominal lymphadenopathy.

Although indicators of inflammation remained normal throughout the course of treatment, the initial clinical presentation was suspicious for infection. Therefore, the manufacturing process of MSCs was reevaluated and contamination by infectious agents during cultivation or before release was ruled out. An expanded diagnostic investigation (Supplementary Appendix and Table 2) ruled out common and opportunistic infections, including Herpes viruses 6, 7, and 8, CMV, HIV, and EBV viremia, bacteremia, gastrointestinal infections, and the possibility of intestinal bacterial overgrowth syndrome. Endoscopic biopsies of the colon and duodenum showed mucosal edema without inflammatory cell infiltration or apoptotic bodies. Given the normal concentrations of complement components and absence of fetal bovine albumin in the cell medium (platelet lysate was used instead), a serum disease-like syndrome did not seem likely. A capillary leak variant syndrome, associated with sudden capillary hyperpermeability, resulting edemas and hypoalbuminemia, was considered. Therefore, antihistamines and methylprednisolone were administered at a dosage of 1.5 mg per kg body weight. After administration of high-dose steroids, the symptoms disappeared, and the dose was reduced during the next 10 days. At this point, sCr stabilized in the range of 300 μmol/l.

**Figure 3:**
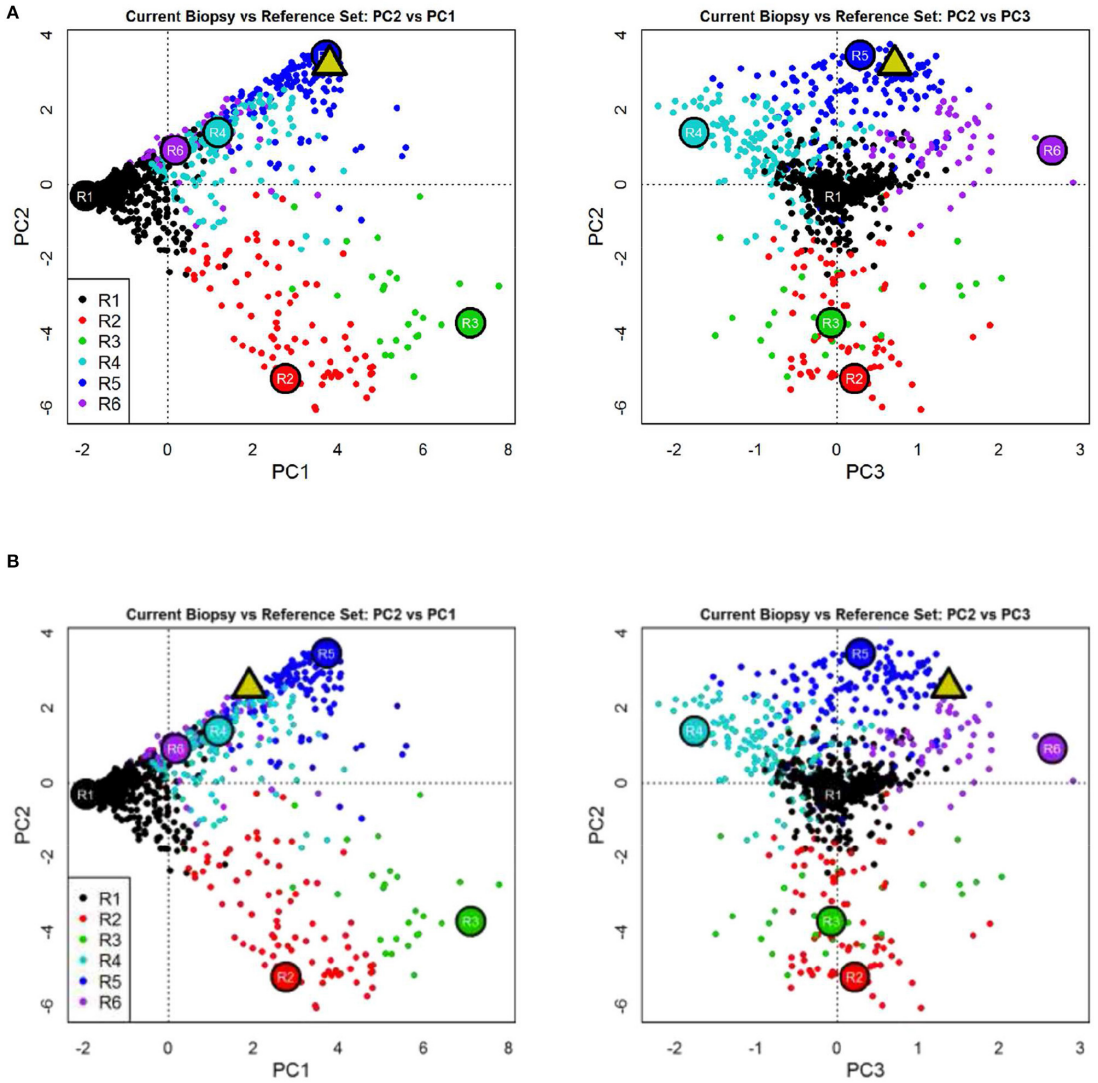
Molecular microscope diagnostic report (MMDx) for kidney transplant biopsy before **(A)** and after **(B)** application of mesenchymal stem cells showing fully developed antibody-mediated rejection with extensive microvascular inflammation, chronic glomerulopathy, and atrophy-fibrosis molecular features.

Two weeks later (i.e., 5 weeks after the last dose of MSCs), there was a recurrence of abdominal symptoms (nausea, diarrhea, ascites), this time followed by resistant hypertension, a further deterioration of kidney function (sCr of 380 μmol/l), an increase in proteinuria (7.3 g per day), and signs of hemolysis and pancytopenia. Tacrolimus trough levels were very low (<2 ng/ml), despite an increase of the dosage and assured adherence to therapy. The abdominal CT scan showed ascites, thickening of the intestinal wall (especially the jejunum), lymphadenopathy and an enlarged spleen. Transcriptomic analysis by the molecular microscope diagnostic system (MMDx) (12) of the subsequent kidney biopsy showed persistence of AMR, although the molecular classifiers of inflammation and AMR were lower compared to the biopsy before application of MSCs (inflammation score of 1.06 vs. 3.19, and AMR score of 0.63 vs. 0.94, respectively; Figure 3). In addition, the MFI levels of DSA decreased and we had no reason to assume a further deterioration of AMR.

Cytokine assessment after MSCs treatment (at the time of onset of symptoms) showed increased levels of IL-2R and IL-8, while other cytokines, including TNF-α and IL-6, were within near normal ranges and not consistent with a cytokine storm (Figure 2B, for details see Supplementary Table 2). Comparative analysis of T and B lymphocyte subsets before and after MSC therapy showed a transient decrease in all lymphocyte subsets with an increase in the ratio of activated T lymphocytes that occurred concomitantly with worsening of symptoms and the appearance of pancytopenia (Figure 2A). Bone marrow examination was consistent with trilineage dysplasia and a secondary myelodysplastic syndrome attributable to potentiated immunosuppressive therapy. It is more likely, however, that the changes in the bone marrow were of reactive origin. Morphologically, there was no evidence of parvovirus B19 infection of the bone marrow.

The kidney allograft failed 2 months after MSCs transplantation and hemodialysis was initiated. After administration of granulocyte colony stimulating factor (G-CSF) and reintroduction of methylprednisolone at a dose of 1 mg/kg, leukopenia improved minimally, and gastrointestinal symptoms worsened after each application of GCS-F. Therefore, the steroids and GCS-F were discontinued. The patient's general condition gradually deteriorated with progressive malaise, vomiting/diarrhea, generalized edema, and laboratory signs of hypoalbuminemia and agranulocytosis. The clinic's expert council made the empirical decision to end a life-threatening situation by removing the failed kidney transplant. After the transplant nephrectomy, the cell counts recovered almost instantly (Figure 2A). At this time, parvovirus B19 viremia was also detected. After treatment with high-dose IVIg, the parvovirus B19 infection resolved. Retrospectively, parvovirus B19 was detected by PCR in both the preimplantation kidney biopsy (6 years before MSC therapy) and in the kidney graft after MSC therapy. The patient is currently on chronic hemodialysis, remains aviremic and no further MSCs associated adverse events were observed during the 24-month follow-up period. Patohistologic analysis of the explanted kidney was consistent with advanced AMR, glomerular and vascular thrombotic microangiopathy (TMA), and extensive tubular injury (Figures 1C-F).

## Discussion

Here, we describe a case report of a serious adverse event associated with autologous bone marrow MSCs in KTR as part of a rescue therapy protocol for chronic AMR. To briefly summarize: following the use of autologous MSCs, the patient developed symptoms and signs of systemic disease, including malaise, nausea, vomiting, blepharitis, diarrhea, ascites, splenomegaly, resistant hypertension, hemolytic anemia, nephrotic range proteinuria with rapid deterioration of kidney function, and pancytopenia. Symptoms developed progressively, responded only initially to steroid treatment, worsened after GCS-F and resolved almost immediately after explantation of the failing kidney. Pathohistological findings in biopsied organs after MSCs therapy included: (i) mucosal edema of the colon and duodenum; (ii) MDS-like changes in bone marrow; (iii) new onset of TMA and severe tubular injury in the kidney allograft, which overlaid the previously observed AMR. To the best of our knowledge, these undesirable outcomes have not yet been reported following transplantation of the MSC line.

So far, 10 clinical studies in KTRs using MSCs have been completed. In six of these studies autologous or donor derived MSCs were injected intravenously or intraarterially before or during surgery or up to 6 months after KTx, in a maximum of two doses up to 10 x 10^6^/kg (2–6, 8). Vanikar et al. (7) co-infused donor adipose derived MSCs together with hematopoietic stem cells in portal circulation prior to living donor kidney transplantation. Two recent studies reported on the use of human umbilical cord derived MSCs applied before or during transplant surgery (9) and third-party MSCs up to day 5 after transplantation (10). No serious adverse reactions have been reported. However, the pilot study of the Remuzzi group reported negative effects when autologous MSCs were used in two KTRs (2). They reported a temporary decrease in kidney function with the histological picture characterized by a low number of infiltrating CD4^+^ and CD8^+^ T cells, B cells and monocytes, but a high number of neutrophils with increased deposition of complement component C3. A considerable number of CD105^+^ CD44^+^ double positive cells (markers co-expressed by MSCs) were also detected in the biopsy, suggesting the recruitment of systemically infused MSCs within the kidney allograft. After administration of steroids, kidney function improved in both patients. In contrast, no adverse effects were reported when MSCs were administered before KTx (4). These clinical cases differ from ours, because the histological findings in our patient's transplanted kidney showed neither infiltration of neutrophils nor infiltration of MSCs (CD105^+^ CD44^+^were absent) or complement deposition. It is important to note that none of the patients in the study by Perico et al. (2) showed multi-organ dysfunction.

Given the systemic clinical presentation and patient history, probable causes such as common infections, contamination of MSC product, progression of AMR, recurrence of ALL and adverse effects of immunosuppressive therapy were initially ruled out. Unfortunately, we were not aware of active parvo B19 infection at the beginning because we performed blood PCR only when the clinical picture of pancytopenia progressed. Later, we confirmed parvo B19 in the preimplantation kidney biopsy, suggesting possible donor transmission, so parvo B19 was present in the transplanted kidney long before MSC therapy. Infections with parvo B19 may be associated with significant morbidity in immunocompromised patients (13). The most frequent manifestations of parvo B19 infection in KTRs are cytopenia's with the dominance of anemia. Collapsing glomerulopathy and TMA have been reported in 10% of cases with dysfunction or failure of the kidney transplant (14). In addition, as reported by Sundin et al. (15) MSCs are among the cells that express the B19 receptor (P-antigen/globoside) and a co-receptor and harbor the parvovirus B19, which can impair their clinical utility. Subsequent analysis of the patient's historical sera and a pretransplant biopsy of the donated kidney revealed that the virus was transmitted to the recipient via a donated organ, and that the parvo B19 viremia was present shortly before MSCs transplantation. Although MSCs did not induce viremia, the cumulative effect of additional immunosuppression inhibited the B-cell response, which may have had an enhancing effect on the replication of parvovirus B19.

The pleiotropic clinical picture with gastrointestinal involvement, massive transudation of plasma into the third space and pancytopenia strongly suggested early complications that would otherwise be expected after hematopoietic stem cell transplantation, in particular capillary leak syndrome (16). In addition, the continuous clinical picture observed in this case showed some similarities with other entities such as engraftment syndrome (17, 18), hemophagocytic lymphohistiocytosis (19), autologous GVHD (20), and POEMS syndrome. However, strict criteria for making one of the mentioned diagnoses were not met. Therefore, it is highly probable that endothelial damage due to the action of various and difficult-to-define transplant-related factors played an important role in its development. The progressive endothelial damage led to a prothrombotic and proinflammatory state in the patient, which eventually resulted in capillary occlusion. Dysregulation at the level of the complement system and/or the possible presence of antibodies, either donor- or recipient-specific, or as yet unrecognized inflammatory triggers could play a significant and crucial role in terminal endothelial damage. In animal studies, Koch et al. (21), who administered isogenic MSCs to rats at the time of allogeneic KTx, reported similar adverse effects. All animals developed severe kidney failure, 20% of which developed TMA, and 75% had to be sacrificed within 30 days. These life-threatening events were evident despite decreased T- and B-cell kidney allograft infiltration, reduced interstitial inflammation and downregulated inflammatory genes.

Many other studies in animal models of KTx emphasized the importance of the timing of MSCs infusion and provided evidence for improved outcomes when MSCs transplantation was performed prior to KTx [for details see review by Casiraghi et al. (22)], with higher renoprotective and anti-inflammatory effects when used prior to the development of a potent inflammatory microenvironment. However, subsequent studies have shown that peri-transplant infusion of syngeneic MSCs can lead to severe kidney failure associated with tissue injury, increased expression of pro-inflammatory cytokines, B-cell infiltration and C4d deposition in the allograft, regardless of the immunosuppressive protocol used (23).

## Conclusion

This case report describes the first clinical case of acute kidney transplant failure with life-threatening systemic adverse effects after autologous MSCs transplantation to reverse late AMR. The scenario described suggests important safety concerns related to the use of MSCs in the long-term period following solid organ transplantation when the allograft is already injured by chronic active rejection.

Considering the absence of similar adverse effects in the two patients who followed the same study protocol (follow-up periods of 24 and 20 months after MSCs application; unpublished results), the individual susceptibility of the patient may also have played a role in the development of adverse events. Nevertheless, for safety reasons, we would strongly recommend further preclinical research before proceeding with clinical trials to accurately assess the benefits and risks of MSC therapy in the context of solid organ transplantation. The current report also highlights the importance of monitoring for possible transmission of parvovirus B19 through MSCs products or donor organs.

## Data Availability Statement

The original contributions presented in the study are included in the article/Supplementary Material, further inquiries can be directed to the corresponding author/s.

## Ethics Statement

The studies involving human participants were reviewed and approved by National Medical Ethics Committee. Štefanova 5 1000 Ljubljana. The patients/participants provided their written informed consent to participate in this study. Written informed consent was obtained from the individual(s) for the publication of any potentially identifiable images or data included in this article.

## Author Contributions

ŽV-H and MA: study design, performance of the research, data analysis, and drafting of the manuscript. NK: pathohistological diagnosis, performance of the research, and manuscript revision. PH: MMDX analysis and critical revision. MS, SZ, and TU: study conception, performance of the research, and critical revision. UŠ, PP, and KH: performance of the research, analysis and interpretation of data, and critical revision. GM, MO, AA, AI, and JB: acquisition and interpretation of data, and critical revision. All authors have approved the final version of the manuscript.

## Conflict of Interest

The authors declare that the research was conducted in the absence of any commercial or financial relationships that could be construed as a potential conflict of interest.
