# Supplementary material for: Case Report: Capillary Leak Syndrome With Kidney Transplant Failure Following Autologous Mesenchymal Stem Cell Therapy
Source: Front Med (Lausanne). 2021 Jul 21;8:708744. doi: 10.3389/fmed.2021.708744 (PMC8334176; doi:10.3389/fmed.2021.708744)
Supplement: Supplementary file 2 [file Data_Sheet_2.docx]

**Supplementary appendix**

**Appendix S1. Characteristics and culture protocol for autologous MSCs**

After signing informed consent patient was referred for bone marrow collection. On day 0, 217 ml of autologous bone marrow (plus ACD, physiological saline and heparin) was seeded in double cell stack plates (CellSTACK 2 chambers, Corning, USA) 0,5 x 10^6^ cells/cm^2^. Bone marrow samples were taken for sterility and mycoplasma testing. Culture media (MEMα, Gibco, USA) was supplemented with platelet lysate (prepared from 5 donations of apheresis platelets, freeze-thaw preparation, Blood Transfusion Centre of Slovenia, Slovenia) to 5% in the final concentration, gentamicin (Garamycin, Krka, Slovenia) 50 µg/ml and heparin (Heparin, B. Braun Mesulgen, Germany) 2 i.u./ml. Primary cells were cultured until 50% confluent. Culture media was changed five times before first trypsinization. On day 21, cells were trypsinized (Trypsin-EDTA, Sigma, USA) and harvested. The yield was 15.200 cells/cm^2^. One third of the cells was reseeded, two thirds were frozen into two freezing bags (CryoMACS Freezing Bags, MACS Miltenyi Biotec, Germany) in 10% DMSO (CryoSure-DMSO, WAK-Chemie Medical, Germany) and human albumin (Albunorm, Octapharma, UK). First infusion was prepared on day 26, when cells were 80% confluent (yield 12.600 cells/cm^2^). After trypsinization cells were centrifuged, rinsed twice with 0.5% human albumin and finally resuspended in 0.5% human albumin. Preparation contained 80 x 10^6^ cells (characteristics are presented in Table S1) in 100 ml of 0.5% human albumin. Samples were taken for phenotype and sterility testing. Cells were also seeded for karyotyping. On day 27, one freezing bag was thawed, and cells seeded. Culture media was changed once. On day 34 (day 7 after seeding), when confluency reached approximately 70% (yield 8.700 cells/cm^2^), the second infusion was prepared. It contained 66 x 10^6^ cells (Table S1). Samples were taken for phenotype and sterility testing. Additionally, cells were seeded for mycoplasma testing and used culture media was sent for endotoxin testing. On day 33, the second freezing bag was thawed, and cells seeded. Culture media was changed once. On day 42 (day 9 after seeding), when confluency reached approximately 90% (19.800 cells/cm^2^), the third infusion was prepared. It contained 74 x 10^6^ cells (Table S1). All MSCs infusions tested negative in sterility testing. Expressed values of CD73, CD 90, CD105 and CD45 were all in acceptable ranges and thus complied to release criteria (Table S1). Cells expressed low levels of HLA-DR. Platelet lysate tested negative for routinely screened markers in blood donors (HBsAg, anti-HCV, anti-HIV I/II and HIV Ag, anti-TP antibodies and NAT for HBV, HCV, HIV) and negative in sterility testing (BAC TEC). There were no deviations from culturing protocol, cells were passaged at confluency 50 to 90%, yields were as expected. We used gentamycin routinely as a preventive measure against microbiological outgrow. As part of regular quality testing, we also performed karyotyping which showed no clonal changes (G-banding). Overall, cell proliferation rate was slower than average. Morphologically, MSCs after first passage appeared to exhibit few more flattened (FC) cells than expected for early culture. The expressed level of CD73 was consistently high and did not suggest senescence. Apart from that cell morphology did not show any unusual features. All three preparations contained cells from second passages, second and third MSC infusion were prepared from thawed and cultured cells. The platelet lysate used as culture media supplement originated from the batch used also for culturing cells for another patient in the same study. This patient experienced no adverse reactions after MSCs infusion.

Criteria for MSCs batch release were the following: expression of CD105, CD73, and CD90 >95%, expression of CD34, CD45, and CD14 <2%; negative for mycoplasma, Gram-positive, and Gram-negative bacteria and fungi; endotoxin below 5 EU/kg; viability >80%; absence of genetic lesions at cytogenetic analysis.

**Table S1.** Infusion characteristics of autologous MSCs.

|  | MSCs infusion 1 | MSCs infusion 2 | MSCs infusion 3 |
| --- | --- | --- | --- |
| Number of MSCs | 80x10^6^ | 66.5x10^6^ | 74x10^6^ |
| Viability (%) | 98.6 | 97.1 | 99 |
| CD105 (%) | 99.7 | 99.2 | 98.3 |
| CD73 (%) | 99 | 99.3 | 97.7 |
| CD90 (%) | 99.9 | 98.7 | 99.7 |
| CD45 (%) | 1.0 | 1.7 | 1.3 |
| HLA DR (%) | 0.4 | 0.1 | 0.4 |

**Table S2.** Results of diagnostic investigations and laboratory findings in the patient.

| **Examination** | | | **Result** |
| --- | --- | --- | --- |
| ***Virology***  Herpesvirus 6  Herpesvirus 7  Herpesvirus 8  EBV  CMV  Hepatitis B  Hepatitis C  HIV 1 and 2  Polyoma BK  Parvovirus B19 | ***Hemocultures***  bacteria, yeast | ***Stool***  Rotavirus Adenovirus Norovirus Astrovirus Yersinia enterocolitica Campylobacter spp. Shigella/enteroinvasive Escherichia coli Salmonella spp. Plesiomonas, stx2 stx1 Giardia Cryptosporidium  E. coli verotoxin vtx1/vtx2  Clostridium difficile toxin, Clostridium difficile culture Tropheryma whipplei  Parasites | all results negative, except for parvovirus B19 |
| ***Serum cytokines concentrations*** *(two time points are presented; values that significantly deviate from the references are marked in red)*  *Reference values:*  *s-IL-2R (soluble IL-2 receptor):158 - 623 U/ml*  *TNF-α: <3.36 pg/ml*  *IFN-γ: < 1.0 pg/ml*  *Interleukin 1 β: <5,70 pg/ml*  *Interleukin 6: <3.98 pg/ml*  *Interleukin 8: <8.91 pg/ml*  *Interleukin 10: <3.42 pg/ml*  *Interleukin 12: <1.53 pg/ml* | | | 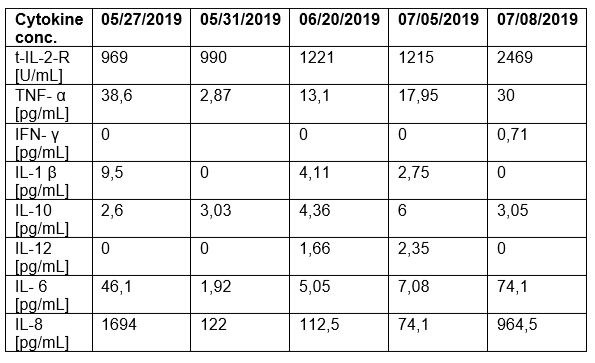  *last (3^rd^) dose of MSCs applied 05/24/2019 |
| ***Basic hematologic parameters***  (only selected dates are presented: 4/11/2019 before MSCs therapy; 5/27/2019, two days after completion of MSCs therapy, when adverse effects occurred; 7/4/2019 on readmission to hospital due to progressive adverse effects; 7/23/2019 on the day before explantation of the kidney allograft). | | | 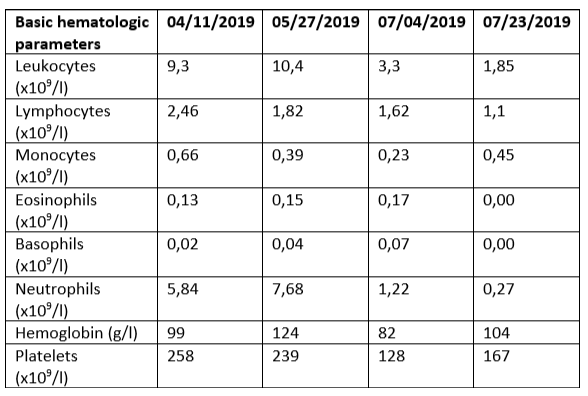 |
| ***Serum complement assesment*** | | | 5/28/2019: activation of the complement system by the classical route - hemolytic test 120%, activation of the complement system by the alternative path: 83E, activation of the complement system by the lectin path: 160% (results within reference limits).  7/5/2019: activation of the complement system by the classical route - hemolytic test: 105%, activation of the complement system by alternative pathway: 71 E, activation of the complement system via the lectin path: 136% (results within ref. limits).  complement component C3: 0.83 g/l (ref. limits for adults 0.90–1.80 g/l)  complement component C4: 0.21 g/l (ref. limits for adults 0.10–0.40 g/l) |
| ***Quantiferon-CMV monitoring*** (5/30/2019) | | | 7.33 IE/ml (reactive); ref. limits: <0.2 IE/ml (non-reactive), ≥0.2 IE/ml (reactive) |
| ***Immunoserology*** (5/30/2019) | | | s-HEP-2, ANA, anti-ENA (antibodies against soluble nuclear antigens Sm,U1RNP,Ro,La,Scl-70,Jo-1,PCNA,PM/Scl, SL, Ku), anti-beta 2 GP, lupus anticoagulants, cardiolipin antibodies, ADAMTS, anti-ADAMTS, anti IgA, anti- thrombocyte antibodies: all negative. |
| ***Abdominal ultrasound*** (7/13/2019) | | | ascites, thickened section of the small intestinal loops (enteritis), enlarged spleen |
| ***Ultrasound-Doppler of the transplanted kidney*** (repeated in more instances) | | | kidney transplant of normal size, slightly more echogenic parenchyma, preserved global and cortical perfusion, global and cortical, normal intrarenal Doppler indices, no signs of renal artery stenosis |
| ***Peritoneal effusion*** (5/28/2019) | | | transudate, no bacterial or yeast growth |
| ***Bone marrow citology*** (7/5/2019) | | | the finding corresponds to normocellular bone marrow with normal number of megakaryocytes, mature eosinophils infiltration (10-15%) without blast cells; the examination is consistent with myelodysplastic syndrome due to the morphological signs of dyseritropoiesis manifested by cell nucleus and cytoplasmic abnormalities, and due to hypogranulation of mature neutrophils |
| ***Bone marrow biopsy*** (7/5/2019) | | | for patient age normal to mild hypocellular marrow in which disorders of cell maturation in all three hematopoietic lines are seen; changes can be due to the set of reactive changes upon administration of immunomodulatory therapies; given the history of treatment of acute lymphoblastic leukemia in childhood it is necessary to think about the possibility of development of myelodysplastic syndrome (with respect to pancytopenia and morphologic changes in bone marrow); comparative to biopsy prior to MSC transplantation, it is the impression that bone marrow is now less cellular, with more suppressed granulopoiesis and erythropoiesis; no ALL infiltrates are observed |
| ***Flow cytometry bone marrow*** (7/5/2019) | | | 2.2% of CD34 positive cells and 25% of lymphocytes with normal lymphocyte T/B ratio were determined in the received specimen; 9% of cells of the monocytic type were identified, the rest were myeloid cells in maturation; infiltration with malignant cells has not been demonstrated |
| ***Esophago-gastro-duodenonoscopy*** (7/18/2019) | | | esophagus: normal mucosa  stomach: the mucous membrane of the corpus is redish, antrum unremarkable  duodenum: normal mucosa  proximal jejunum: normal mucosa |
| ***Duodenal aspirate*** (7/18/2019) | | | aerobic and anaerobic microorganisms:  <10^5^ CFU/ml (normal)  Giardia: negative |
| ***Gastric and duodenal biopsy*** (7/12/2019) | | | A. duodenum, ascendent part:  duodenitis chronica exigua, hyperplasia glandularum Brunneri levis, histological changes correspond to mild peptic duodenitis, the duodenal mucosa has no significant changes, suggestive for GVHD (no apoptosis); negative immunohistochemical staining for CMV  B. stomach:  gastritis chronica levis, fibrosis laminae propriae focalis levis, H.Pylori neg.; changes correspond to mild reactive (chemical) gastritis with small chronic erosion in the antrum; negative immunohistochemical staining for CMV; there are no changes suggestive for GVHD (no apoptosis found, mucous membrane with signs of mild reactive gastritis) |
| ***Partial colonoscopy*** (9/7/2019) | | | partial colonoscopy to sigmod colon, depth 25 cm: endoscopically normal appearance of sigmoid mucosa, no erosions |
| ***Rectal biopsy*** (5/29/2019; 7/12/2019) | | | rectum: proctitis chronica levis, edema laminae propriae, rectal mucosa samples have slight changes (slightly lower mucosa,focally resolved crypts, probably due to lamina propria edema, rare lymphocytes in lamina propria); individual apoptosis in crypt epithelium (up to a maximum of 3 in a 2 mm long specimen); no erosions; histological changes are mild and unremarkable and cannot be etiologically specified; individual rare apoptosis in the crypt epithelium indicate the possibility of GVHD, but are not diagnostic (rare apoptosis may be due to other causes - medications, colonoscopic preparation); immunohistochemically negative for CMV |
| ***Abdominal computer tomography*** (7/16/2019) | | | ascites; no signs of bowel ischemia; visceral vessels well transient; the jejunal wall is diffusely thickened (up to 8 mm) – enteritis; in the surrounding area of small intestine multiplied and enlarged mesenteric lymph nodes are visible (up to 5 mm); native kidneys are atrophic; transplanted kidney is of normal length; the liver, gallbladder, bile ducts, spleen, pancreas, bladder, adrenals are within normal limits |
| ***PET-CT scan*** (7/16/2019) | | | moderately elevated intestinal metabolic activity may be physiological, inflammatory events are less likely; somewhat inhomogeneously diluted bone in the left lamina of vertebra C6, the 5th left rib, and in the wing of the right intestine, where radiopharmaceutical uptake is not elevated; elsewhere in the body shown there are no scintigraphy signs of a metabolically active malignant process |
| ***Donor specific anti-HLA antibodies*** (Luminex) | | | 4/11/2019: anti-DQB1 (MFI 3700), anti-DQA1 (MFI 4000)  30/5/2019: anti-DQB1 (MFI 3390), anti-DQA1 (MFI 2520)  7/8/2019: anti-DQB1 (MFI 3230), anti-DQA1 (MFI 2530) |
| ***Anti-AT1R antibodies*** (4/12/2019) | | | negative |
| ***Autologous anti-HLA antibodies*** (7/8/2019) | | | negative |
| ***Autologous antineutrophil antibodies*** (7/8/2019) | | | negative |
| ***Mixed chimerism*** (7/8/2019) | | | absent |
